# Greener Microplastics Removal: Progressive Replacement of Iron‐Based Coagulants with Sodium Alginate and Chitosan to Enhance Sustainability

**DOI:** 10.1002/cplu.202400736

**Published:** 2025-02-26

**Authors:** Marco Facchino, Loris Pietrelli, Patrizia Menegoni, Mauro Capocelli, Emanuele Limiti, Marcella Trombetta, Francesco Basoli, Marcello De Falco

**Affiliations:** ^1^ Department of Science and Technology for Sustainable Development and One Health Università Campus Bio-Medico di Roma Via Álvaro del Portillo, 21 00128 Rome Italy; ^2^ Scientific Committee Legambiente Via Salaria 403 00199 Rome Italy; ^3^ CR Casaccia ENEA Via Anguillarese, 301 Rome Italy

**Keywords:** Microplastics removal, Enhanced coagulation, Natural coagulants, Charge neutralization, Adsorption

## Abstract

Wastewater treatment plants (WWTPs) currently represent one of the main sources for microplastics (MPs) and other emerging contaminants entering the environment. Coagulation is a longstanding and cost‐effective process designed to enhance the removal of colloidal particles and proved to be efficient in the abatement of MPs. The present study investigates the feasibility of a progressive replacement of ferric chloride (FeCl_3_) with chitosan (CT) and sodium alginate (SA), starting from their use as coagulant aids. Coagulations tests were carried out to assess the performance of FeCl_3_‐CT and FeCl_3_‐SA systems in the removal of polystyrene (PS) microbeads, polyethylene (PE) and polyethylene terephthalate (PET) fragments with sizes lower than 500 μm. Results from experiments have shown that both CT and SA are useful to enhance the removal performance of conventional coagulation by improving the settling characteristics of flocs. The use of CT allows a reduction of coagulant dosage for removing PS and PE particles, while it turned out to be detrimental for the removal of PET fragments. Instead, SA at a concentration of 0.2 mg L^−1^ proved to be useful both to achieve higher removal rate at a medium dosage of coagulant and to improve the efficiency of the process at lower dosages.

## Graphical abstract

**Delve into the use of natural coagulants to find sustainable alternatives for the removal of microplastics from wastewater**. Chitosan and sodium alginate were used in our study to investigate the potential of a transition away from inorganic salts in wastewater treatment. The results pave the way for more environmentally friendly and cost‐effective solutions to manage emerging contaminants in water systems.

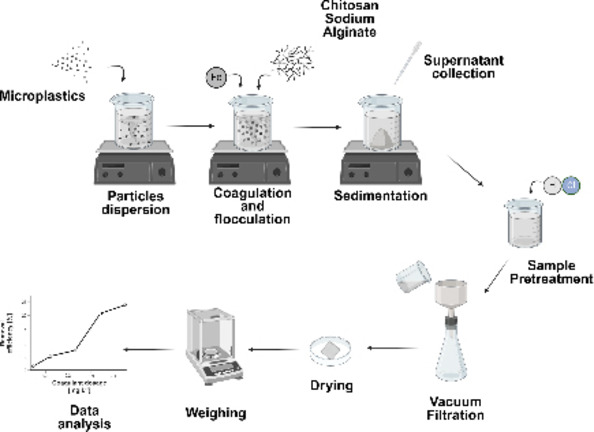

## Introduction

The massive adoption of plastic materials undeniably transformed human life and enhanced its quality.[^1^, ^2^] The same properties that enabled their penetration into a plethora of sectors are currently the reason behind plastic pollution and its threat to both environmental and public health.^[3]^ First raised in 1972 with the finding of 500 μm polystyrene (PS) spherules in the coastal waters of southern New England,^[4]^ the issue of plastic pollution became even more alarming after the identification of small debris with sizes down to 20 μm, labelled microplastics (MPs), available for ingestion from marine biota.^[5]^ To date, up to 80 % of marine litter consists of plastic,^[6]^ with almost 80 % of plastic debris found in the marine environment being linked to a land origin and around 18 % being related to sea‐based activities.[^7^, ^8^] For decades, there was no consensus among the scientific community upon the actual definition of MPs,[^9^, ^10^] which are currently defined from the European Chemical Agency (ECHA) as “synthetic polymer microparticles” whose dimensions are either “equal or less than 5 mm” or whose length “is equal or less than 15 mm and their length to diameter ratio is greater than 3”, to account also for fibers.^[11]^ The ubiquitous presence of MPs in aquatic environments^[12]^ is mostly favored by their small sizes and persistence,^[13]^ while their distribution is mostly affected by both the conditions of the surroundings and the nature and quantity of components sorbed onto the surface.[^14^, ^15^] The threats behind this massive MPs contamination are connected to the sorption and transport capacity of other hazardous pollutants^[16]^ and to the ease of uptake by organisms at the low level of the trophic system.[^17^, ^18^, ^19^] Besides, due to the transfer to humans through the food chain,[^20^, ^21^] there is also increasing evidence of the presence of MPs in the human body,[^22^, ^23^] with recent studies pointing out the potential for increased risk of cardiovascular diseases caused by the interaction of MPs with the organism.^[24]^ To curb plastic pollution, the European Union (EU) recently updated its policy framework related to the design and management of plastic materials, setting the ambitious objective of reducing microplastic releases to the environment by 30 % by 2030.^[25]^ First, since according to the estimates nearly 28 % of the MPs intentionally employed in a diverse range of products (i. e., personal care products, detergents, painting, etc.) end up in the environment, their use was restricted for products intended for the EU market.^[11]^ However, these so‐called primary microplastics represent just a portion of the global challenge,^[26]^ since several of the MPs found in the environment are of secondary nature, meaning that their formation is linked to the synchronous action of physical forces, biological and chemical agents, and photodegradation over larger plastic items.[^27^, ^28^] Therefore, a holistic and efficient strategy must entail eco‐design and proper waste management practices[^29^, ^30^] along with a more in‐depth monitoring of the source of MPs pollution. Among these latter, freshwater systems represent the major routes for MPs entering the aquatic environment,^[31]^ mostly due to rivers being the sink of stormwater runoff and of the effluents of wastewater treatment plants (WWTPs).[^32^, ^33^] WWTPs are indeed increasingly acknowledged as sources and collectors of MPs[^34^, ^35^, ^36^, ^37^] and could be regarded as the last hurdles for preventing the release of MPs in the environment, estimated to account to 3.85×10^16^ MPs per year.^[38]^ Current WWTPs are not designed to target the removal of MPs and other emerging micropollutants,[^39^, ^40^] and this mandates a thorough revision of the current design and deployment.^[41]^ Among the several treatment methods investigated and tested, coagulation‐flocculation‐sedimentation (CFS) has been increasingly reported as critical contributor in the removal of micropollutants.[^42^, ^43^] Hidayaturrahman & Lee found out that the removal efficiency of MPs in facilities equipped with a CFS unit reached rates up to 81.6 % when considering the secondary effluent.^[44]^ The performed experimental campaigns at laboratory scale also showed promising results, with estimated removal efficiencies mostly overcoming 90 % and, prospectively, significant room for innovations securing the removal of these contaminants while comprehensively enhancing the sustainability performance of the CFS processes.^[45]^ Indeed, while conventional inorganic salt coagulants based on iron (such as ferric chloride, FeCl_3_) and aluminum (such as poly aluminum chloride, PAC) led to removal rates above 99 %,^[46]^ these chemicals are also associated with substantial sludge generation and secondary pollution.^[47]^ To this end, the progressive replacement of conventional inorganic salt coagulants with organic and natural coagulants is seen as the most feasible route to guarantee safety, cost‐effectiveness, and environmental mitigation.[^48^, ^49^, ^50^] Among the organic compounds, polyamines were widely tested as coagulant aids.[^51^, ^52^, ^53^] Nonetheless, natural coagulants are regarded as the most promising candidates given the higher degree of biocompatibility, safety, and biodegradability.[^45^, ^47^] Chitosan (CT) and sodium alginate (SA) both represent a potential solution in this field. CT, the second most abundant biopolymer in nature after cellulose, obtained from deacetylation of chitin, has been applied for the removal of both conventional and emerging contaminants from wastewater.[^54^, ^55^, ^56^] Targeting MPs, Raji et al. investigated the feasibility of using a FeCl_3_‐CT system to promote the coagulation‐led removal of 1 μm PS microbeads in a synthetic water matrix, obtaining rates up to 99.8 % at favorable conditions,^[57]^ while Park et al. successfully performed a surface modification treatment of microbeads using tannic acid‐chitosan conjugates to leverage the metal–phenolic coordination bonds.^[58]^ The adopted procedure demonstrated the effectiveness of the method to obtain removal rates higher than 80 % for polyethylene (PE), PS and polymethyl methacrylate (PMMA) beads below 150 μm. All tested polymers in case of FeCl3‐promoted coagulation.^[59]^ Similarly, SA is a widely distributed polysaccharide obtained from the cell walls of brown seaweed which led to promising results in the treatment of turbid water[^60^, ^61^] and as coagulant aid for the removal of MPs. Zhang et al. demonstrated the increase in removal rates led using SA as aid for PAC coagulation systems targeting PET fragments (100–400 μm),^[62]^ whereas Huang et al. reached removal rates higher than 80 % for both PAC‐CT and PAC‐SA systems targeting 100–300 μm polyethylene terephthalate (PET) MPs.^[63]^

Based on the literature findings, the present study evaluates the effectiveness of CT and SA as coagulant aids in CFS treatment for the removal of MPs, using FeCl₃ as the primary coagulant. While current research trends advocate for the full substitution of inorganic salts, such an approach may be technologically premature given the cost, availability, and competing demands of the agro‐industrial and food sectors. By exploring a progressive replacement strategy, this study provides a pragmatic approach to exploit the synergy between natural and inorganic coagulants, supporting the transition toward sustainable WWTP management while being consistent with eco‐friendly treatment strategies and evolving environmental policies.

## Materials and Methods

### Coagulants

Ferric chloride hexahydrate (FeCl_3_⋅6H_2_O), used as primary coagulant, was purchased from Carlo Erba Reagents. CT (75–85 % degree of deacetylation, 190,000–310,000 Daltons) and SA as coagulant aids, hydrochloric acid (HCl), and sodium hydroxide (NaOH) for pH adjustment were purchased from Sigma‐Aldrich. Stock solutions of the coagulant (10 mg L^−1^) were prepared weekly, while coagulant aids solutions (1 % w/v for both CT and SA) were prepared daily to avoid aging or deterioration. Stock solutions were prepared using deionized water (characteristics in terms of conductivity) and kept at 4 °C in the dark. The chemicals and reagents used in the study were of analytical grade and were used without any purification.

### Microplastics

**Figure 1:**
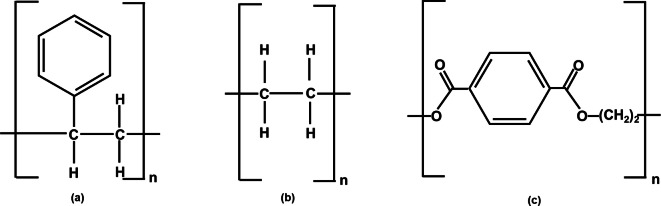
Monomers of the selected MPs: a) PS; b) PE; and c) PET.

PS microbeads (Dynoseeds® TS) with a 40 μm mean diameter were used as sample of pristine spherical samples, whereas PE and PET fragments were obtained by the crushing of commercial water bottles and bottle caps. Figure 1 depicts the monomers of the selected MPs. PE and PET fragments were prepared based on the cryomilling procedures reported in literature[^64^, ^65^] and adapted in‐house. The cryogenic grinding was performed by cooling down the coarse fragments of PE and PET at −80 °C to embrittle the structure and enhance the subsequent crashing performance. Our in‐house adaptation also included pouring distilled water into the blender to avoid reaching the melting point of the polymers. The freezing and crushing procedures were repeated three times. Once obtained, the mixtures made up of small fragments of PE and PET were filtered through three stainless steel sieves with mesh sizes of 3000 μm, 1000 μm, and 500 μm. The filtrates of the last mesh were collected and vacuum filtered (20 μm pore size filter paper) to obtain fragments with sizes in the range 20–500 μm. The obtained fragments were air‐dried and characterized by means of Fourier Transform Infrared Spectroscopy (FT‐IR) (Carry 630 FTIR, Agilent). The morphological and dimensional characteristics of MPs’ samples were verified using an optical microscope (ECLIPSE Ti, Nikon). PS microbeads were equally characterized.

### Coagulation‐Flocculation Experiments and Measurement of MPs

The CFS experiments were performed using a six‐plate magnetic multi‐stirrer (Velp Scientifica, Italy) and 200 mL beakers filled with 100 mL of deionized water at an initial pH of 6 and ambient temperature.

A precision balance with a minimum range of 1.0×10^−5^ g (Sartorius, Germany) was used to weigh MPs before spiking them into the solution to ensure an initial concentration of 300 mg L^−1^. To ensure a proper distribution of MPs, the suspensions were intensively agitated for 3 minutes at 700 rpm before the test began. Prior to performing the coagulation experiments, three blank tests for each polymer were conducted to investigate the free sedimentation behavior of MPs. The first batch of tests was performed to investigate the free‐sedimentation behavior of the MPs suspension in a range of pH from acidic conditions (pH 4) to basic conditions (pH 9). Then, all tests were performed at pH 7. FeCl_3_ was added after microplastics dispersion, together with a predetermined amount of 1 M HCl or NaOH to adjust pH and ensure the formation of the hydroxides.^[66]^ The stirring speed was maintained at 500 rpm for 1 minute after the injection of the primary coagulant, then decreased to 90 rpm for 15 minutes. In the case of FeCl_3_‐CT and FeCl_3_‐SA tests, coagulant aids were added 30 seconds after FeCl_3_ injection. At the end of the flocculation phase, the system was left to settle for 30 minutes, covered to avoid atmospheric contamination. The removal efficiency was estimated using the weighing method as a quantitative approach,[^52^, ^62^] given its cost‐effectiveness and accuracy in presence of simple aqueous matrices.^[45]^ After sedimentation, an aliquot of the supernatant was carefully taken using a syringe and poured into a smaller beaker, where 1 M HCl was used to dissolve the residual flocs from the surface of the MPs. Then, the suspension was vacuum filtered over a priorly weighted 20 μm cellulose filter paper (Stony Lab, 46∅) to separate the MPs, which were subsequently left to dry for 24 hours at room temperature. Eventually, the mass of the MPs and the filter paper was registered and the final concentration ($C_{fin}$ ) was retrieved. The removal efficiencies were benchmarked with the final concentration of the free‐sedimentation tests ($C_{in}$ ) and therefore computed according to the formula below.

$$Removal\;efficiency\;\left(\%\right)=\frac{C_{in}-C_{fin}}{C_{in}}\cdot 100$$

**Figure 2:**
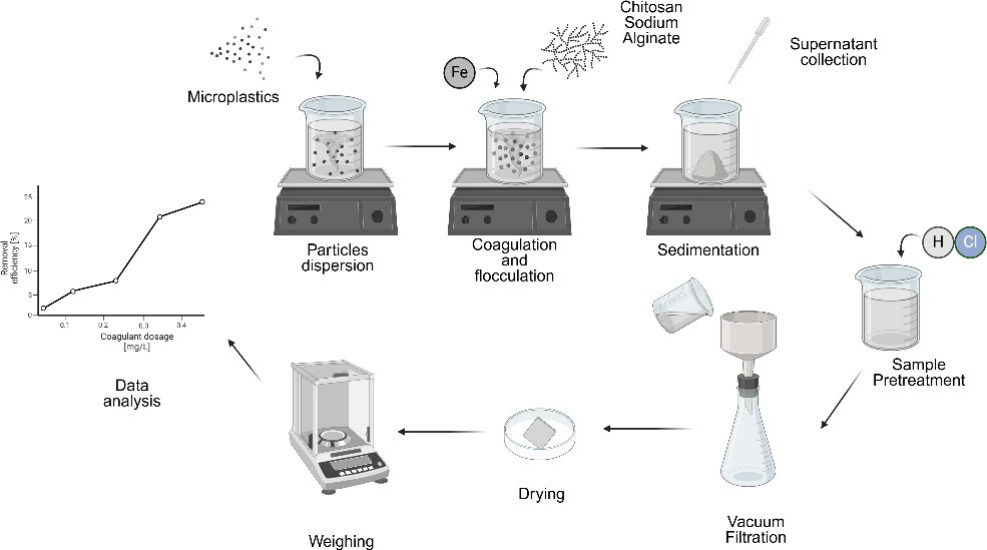
Schematic representation of the adopted procedure for CFS experiments. (Created in BioRender. Facchino, M. (2025) https://BioRender.com/m33k591).

To ensure the repeatability of the results, each test was performed three times. Figure 2 depicts a schematic illustration of the complete procedure.

### Characterization of Supernatants and Sediments

The performed tests included the assessment of the ζ potentials by means of Zeta Sizer Ultra (Malvern, United Kingdom) to investigate the colloidal behavior of the MPs in the suspension and the contribution of charge neutralization of the effectiveness of the CFS process. The ζ potential of the supernatant was indeed measured to obtain insights into the changes in the electrophoretic mobility of MPs before and after coagulation. The collected sediment was lyophilized for 48 hours to obtain a water‐free dried powder. FT‐IR spectra in the 3800–600 cm^−1^ range, with 124 scans and 4 cm^−1^ resolution were acquired to identify the functional groups of the sediment and investigate the contribution of adsorption phenomena.

### Statistical Relevance

A thorough comparison of the results in terms of removal efficiency under different treatment conditions was obtained by performing one‐way and two‐way ANOVA tests followed by Tukey's multiple comparison. The analyses were conducted using GraphPad Prism (version 10.4.1). The tests were used to assess the effects of two independent factors, namely the dosage of FeCl₃ and the dosages of each coagulant aid used. The interaction between these factors was also examined to determine whether the combined effect of FeCl₃ and CT/SA significantly influenced removal efficiency. The ANOVA tests were conducted using the mean values and standard deviations obtained from experimental replicates. A 95 % confidence interval was applied, with a significance level of 0.05. A p‐value <0.03 was considered to indicate a statistically significant difference, while a p‐value <0.0001 indicated a highly significant difference.

## Results and Discussion

### MPs Characteristics

**Figure 3:**
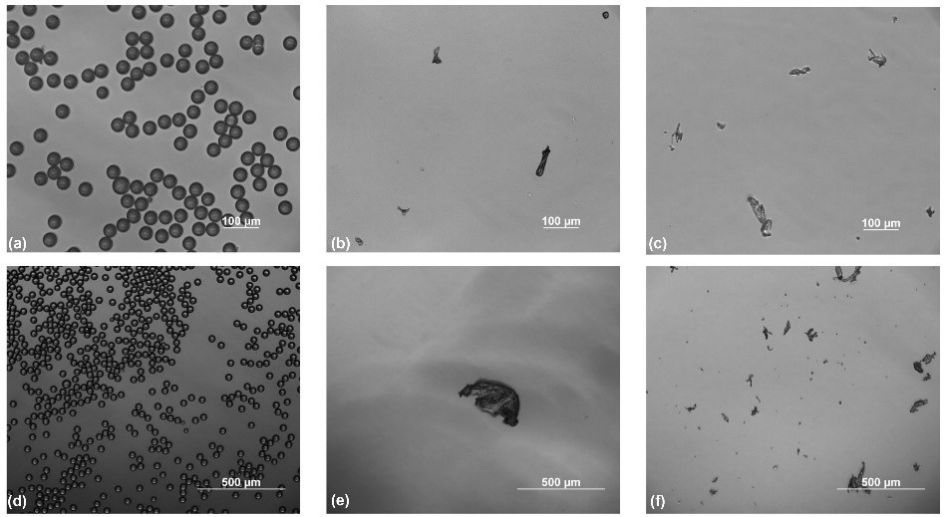
Optical microscopic images of suspended PS microbeads at a) 20x magnification and d) 10x magnification; suspended PE fragments at b) 20x magnification and e) 10x magnification; and suspended PET fragments at c) 20x magnification and f) 10x magnification.

In CFS tests, most studies focused their attention on the entire dimensional range related to MPs (i. e., down to 5 mm,[^51^, ^52^] though some studies focus their attention on particle size lower than 500 μm.[^46^, ^53^, ^62^, ^67^] This size span is the most relevant to address. The outcomes of one of the latest studies on the status of WWTPs effluents pointed out that the smallest particles were most likely to be found in the effluents.^[68]^ Figure 3 reports the images taken at the optical microscopy confirming both the declared size of the PS microbead and the effectiveness of the procedure adopted for PE and PET fragmentation. As can be seen from Figure 3e and Figure 3f, both PE and PET fragments present in the aqueous suspension had sizes lower than 500 μm, with some fragments having sizes comparable to the ones of PS microbeads as shown in Figure 3b and Figure 3c. As for the polymer type, PE and PS are among the most studied MPs being both frequently found in sewage and treatment plants effluents.^[69]^ PE accounts for more than 26 % of the global plastic production^[70]^ and, given its density (0.92–0.97 g cm^−3^), PE particles are likely to be suspended or float in water, even if they were also found in deep sediment.^[10]^ PS, even if less abundant in terms of consumption, is one of the main materials of intentionally added MPs and was widely tested because of toxic‐related issues.[^58^, ^71^, ^72^] Being its density (1.05 g cm^−3^) slightly greater than that of water, PS particles are supposed to settle easier than PE ones. PET has one of the highest densities among plastic materials (1.38 g cm^−3^), and this should enhance its settling. Nonetheless, PET is one of the most detected polymer fibers both in raw and treated water due to its extensive use in the textile industry and the issues related to the discharge of domestic washing machines.[^73^, ^74^]

### Free Sedimentation Behavior of MPs

**Figure 4:**
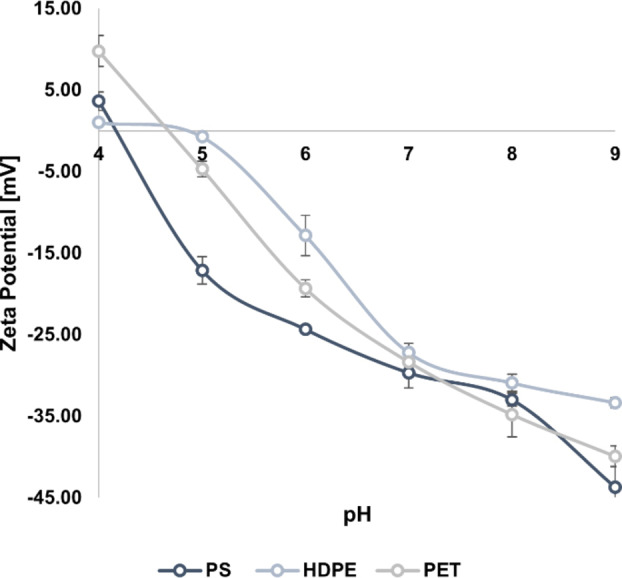
ζ potentials of PS, PE, and PET microplastics at pH ranging from 4 to 9. Error bars represent SD from three independent replicates.

In free‐sedimentation conditions (i. e., without coagulant injection) at pH 7, the residual concentration of PS microbeads was the highest (201.43±6.47 mg L^−1^, 32.86 % abatement), whereas that of PE and PET fragments were of 75.27±5.01 mg L^−1^ (74.91 % abatement) and 27.96±0.77 mg L^−1^ (90.68 % abatement), respectively. This is coherent with the findings of Zhou et al., reporting a 3.19 % abatement of PE particles versus a 50.78 % removal of PS fragments of similar size (less than 500 μm), which the authors ascribed to the different densities of PE and PS.^[67]^ Similarly, Zhang et al. investigated the self‐sinking tendency of PET fragments reporting that nearly 30 % of the particles settled in the absence of the coagulants.^[62]^ Besides the tendency to sink and float related to the material density, it must be noted that MPs are thought to act as colloids^[75]^ and their colloidal behavior is supposed to be regulated by the DLVO theory.^[76]^ Therefore, to investigate this behavior, the free‐sedimentation tests were performed in a broader range of pH, and it was found that free‐sedimentation was strongly influenced. At pH 4, the residual concentrations of PS, PE, and PET were the lowest, with values of 59.60±19.05 mg L^−1^, 43.34±11.93 mg L^−1^, and 14.03±8.81 mg L^−1^, respectively. Raising pH up to 9, the free‐sedimentation tendency strongly decreased, with residual concentration of PS, PE, and PET increasing to 239.43±23.17 mg L^−1^, 158.69±10.36 mg L^−1^, and 48.40±8.35 mg L^−1^, respectively. The assessment of the ζ potential values confirmed the colloidal behavior of the MPs in the sample matrix. Indeed, as reported in Figure 4, for all the investigated particles the ζ potential was steadily negative from pH 4–5, consistently with literature findings reporting the negative charge on the surface of MPs becoming more pronounced with increasing pH for the effect of hydroxyl ions deposition.^[77]^ Based on the obtained data, CFS processes could potentially perform better in acidic conditions.^[51]^ However, to stay aligned with the retrieved literature and the normal operating conditions of WWTPs, pH was maintained around 7 in the tests discussed below.

### FeCl_3_ Coagulation Performance

**Figure 5:**
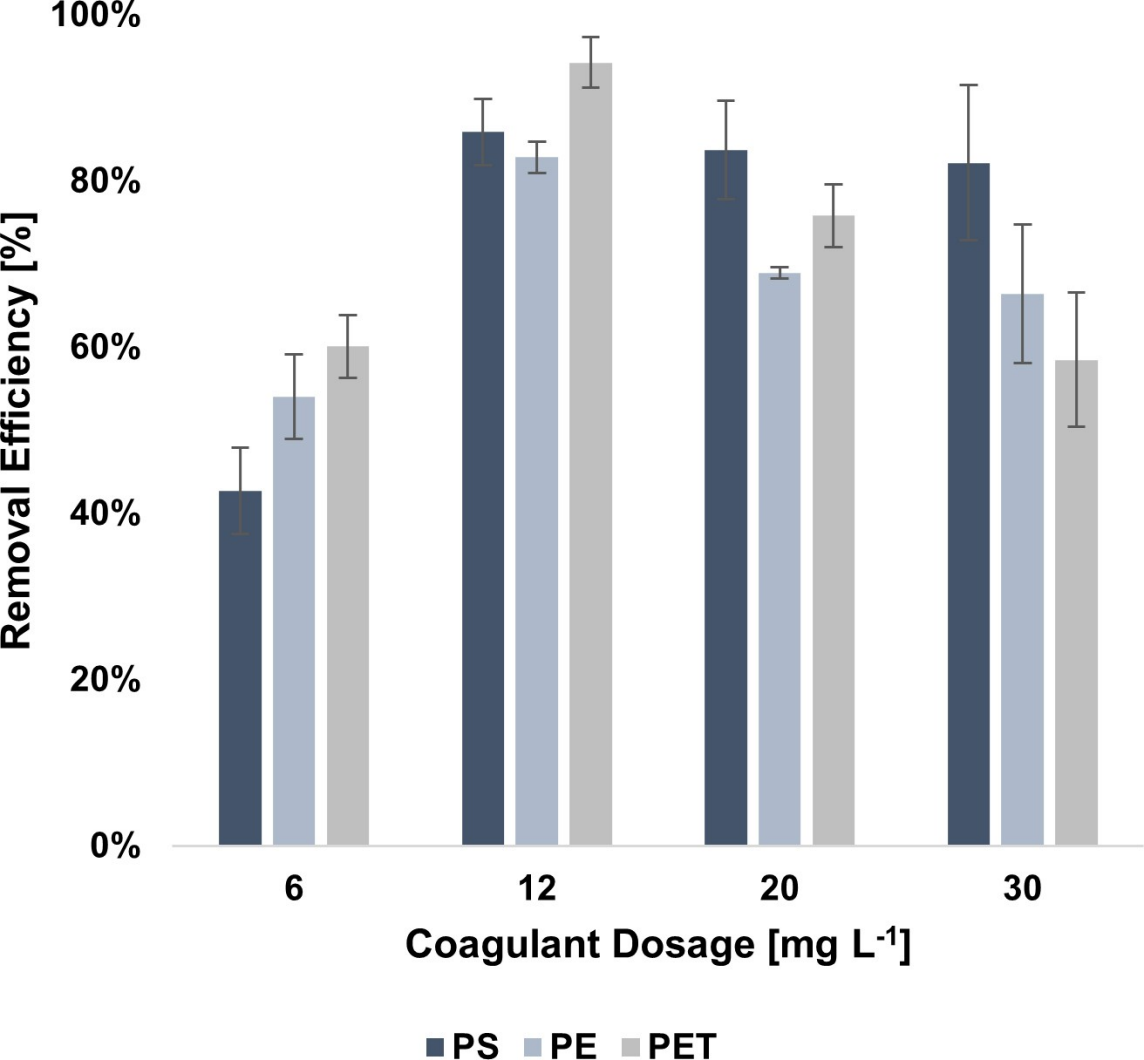
Removal efficiency of PS, PE, and PET microplastics under different FeCl_3_ dosages at pH 7 and initial concentration of 300 mg L^−1^. Error bars represent SD from three independent replicates.

The removal performance of PS, PE, and PET MPs solely using FeCl_3_ as coagulant were investigated at dosages ranging from 6 mg L^−1^ to 30 mg L^−1^ (on iron base), in compliance with the common practice adopted for the dosages of Fe that are typically lower than 20 mg L^−1^.^[78]^ The removal efficiencies of the MPs particles at varying dosages are depicted in Figure 5. The use of a Fe‐based coagulant resulted significantly effective in promoting the removal of all three investigated polymers. useful in enhancing the removal of all three polymers. The trends for all three polymers are characterised by the presence of a maximum at a coagulant dosage of 12 mg L^−1^. The corresponding removal efficiencies for PS, PE and PET were 85.93 %±3.98 %, 82.86 %±1.88 %, and 94.28 %±3.03 %, respectively. For all the investigated MPs, the removal rates slightly decrease going beyond the optimal dosage, with the most marked worsening of performance highlighted for PET, which removal collapse to 58.51 %±8.09 % at a dosage of 30 mg L^−1^.

The statistical analysis revealed that the differences in MP removal efficiency were highly significant (p <0.0001) when increasing FeCl₃ dosage from 6 mg L^−1^ to 12 mg L^−1^ for all three MP types, confirming a strong improvement in coagulation performance at this level. On the other hand, no significant difference was observed for PS when further increasing the dosage of FeCl_3_ beyond 12 mg L^−1^, suggesting a saturation effect. In contrast, higher FeCl_3_ dosages significantly worsened the performances for PE (12 mg L^−1^ vs 30 mg L^−1^, p=0.0477) and PET (12 mg L^−1^ vs 20 mg L^−1^, p=0.0177; 12 mg L^−1^ vs 30 mg L^−1^, p<0.0001). These results confirm that 12 mg L^−1^ represents the optimal FeCl₃ dosage for maximizing MP removal across all tested polymers while avoiding performance decline at higher concentrations. The obtained trends are consistent with the findings of other authors. Ma et al. reported a plateau‐trend for the removal of PE particles with a size lower than 500 μm, highlighting an increase in removal rates from 3.43 % to 12.65 % passing from 5.58 to 279.25 mg L^−1^ of FeCl_3_. The authors also noticed that efficiency slightly decreased increasing the dosage beyond 111.70 mg L^−1^.^[51]^ Zhou et al. obtained an increase in removal efficiency of MPs while raising the FeCl_3_ dosage from 30 mg L^−1^ to 180 mg L^−1^, highlighting that the removal performance remained stable or slightly diminished beyond a dosage of 60 mg L^−1^ and 90 mg L^−1^ for PS and PE, respectively, possibly because the loose structure of the flocs in case of excessive coagulant addition.^[67]^ Further insights on the removal process were obtained through the assessment of the ζ potentials of the supernatants. Indeed, a reduction in the ζ potential value could be an indicator of the compression of the electrostatic double layer and, therefore, of a greater chance of particle collision, aggregation, and subsequent settling.^[79]^

**Figure 6:**
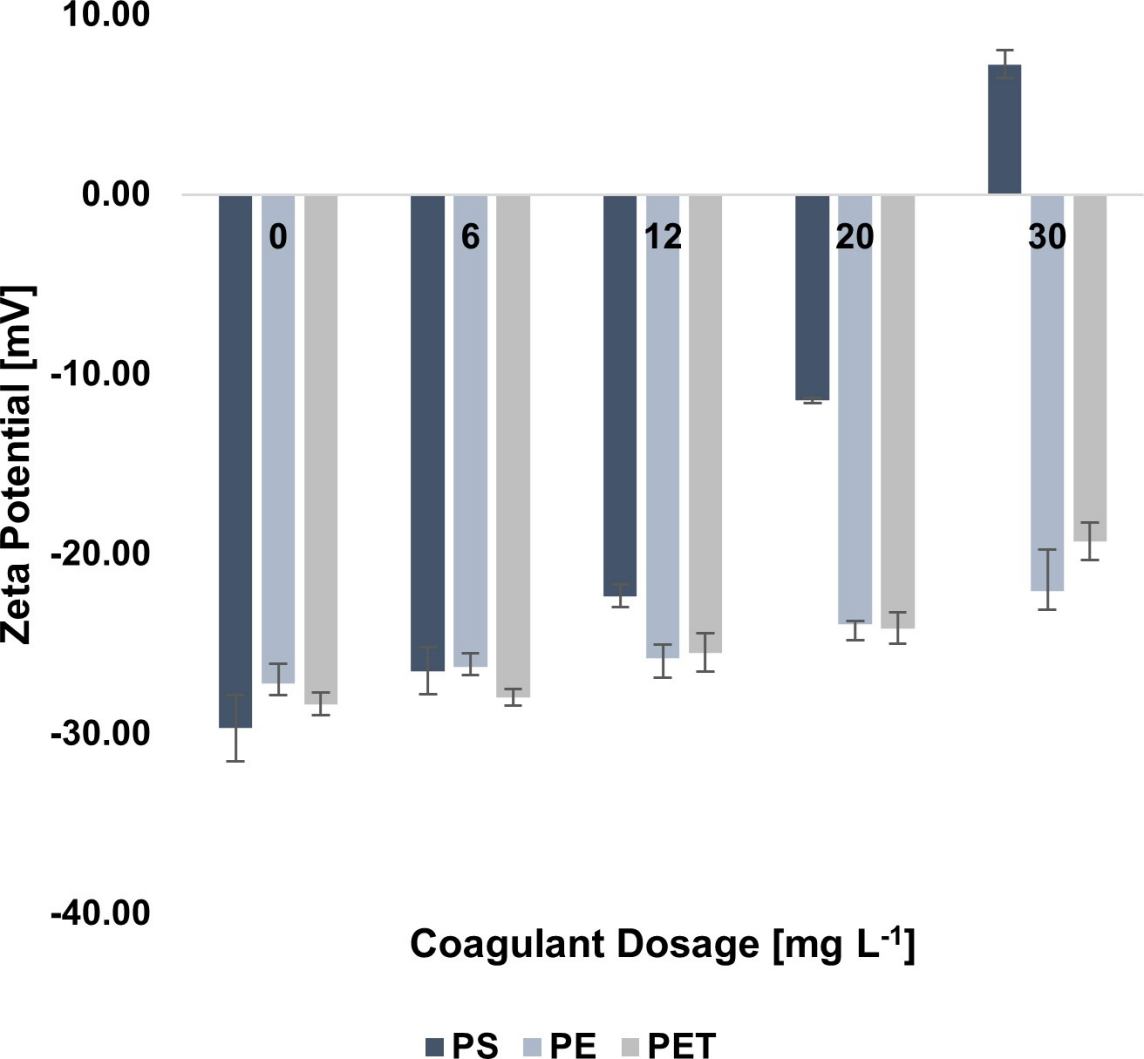
ζ potentials of PS, PE, and PET microplastics in the supernatant after FeCl_3_ led coagulation. Error bars represent SD from three independent replicates.

As shown in Figure 6, a decrease in the absolute value of ζ potential of MPs with respect to the initial conditions occurred, confirming the contribution of the charge neutralization mechanism in the destabilization of the suspension of MPs.

**Figure 7:**
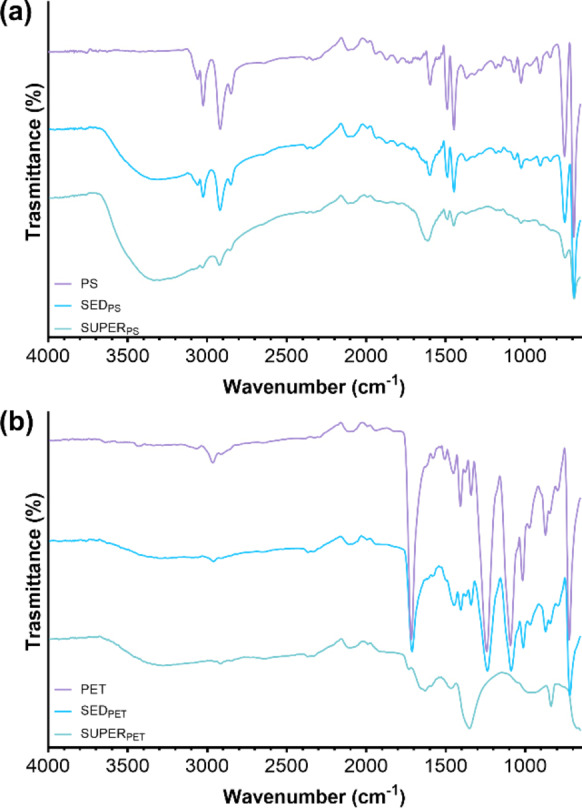
FTIR spectra of a) PS microbeads before testing, sediment and supernatant after FeCl_3_ coagulation, b) PET fragments obtained by cryo‐fragmentation procedure, sediment and supernatant after FeCl_3_ coagulation.

However, the change in ζ potential were significant only in the case of PS microbeads, for which charge reversal also occurred at the highest dosage of coagulant (i. e., 30 mg L^−1^), leading to a positive value of 7.24±0.78 mV. PE and PET fragments did not experience a substantial change in ζ potential, which could have been an explanation for the decrease in removal performance beyond the FeCl_3_ of 12 mg L^−1^. Indeed, the ζ potentials of PS, PE, and PET at that dosage were −22.33±0.64 mV, −25.80±0.74 mV, and −25.48±1.06 mV, respectively, and these were not the values closest to zero among the ones computed. Investigating the removal of PS and PE MPs, Zhou et al. found that charge neutralization occurred for both in the FeCl_3_ coagulation system, reporting a the ζ potentials increase from −15.77 mV and −14.55 mV to −0.57 mV and −7.76 mV, respectively, with a stronger effect obtained in the case of PS removal.^[67]^ On the other hand, Rajala et al. emphasized the absence of a clear correlation between the removal of PS microbeads and the effectiveness of FeCl_3_ as coagulant.^[46]^ Indeed, besides charge neutralization, other mechanisms such adsorption and sweep flocculation could also occur and contribute to the removal process.[^47^, ^80^] The contribution of adsorption was therefore assessed based on the outcomes of the FTIR analyses, performed for both PS and PET to investigate the coagulation mechanisms based on the diverse particle shapes (i. e., microbeads or fragments, respectively). Figure 7 illustrates the IR spectra of the selected polymers (PS and PET), and of the sedimented and supernatant residues of the coagulation/flocculation process for each sample.

In Figure 7a the signals at 3061.8 and 3028.7 cm^−1^ were ascribed to the characteristic aromatic C−H stretching vibrations, and the 2917.0 and 2846.7 cm^−1^ were related to the methylene groups (CH_2_) vibrations. The aromatic C=C stretching peaks can be observed at 1597.3, 1489.8, and 1448.4 cm^−1^, demonstrating the presence of benzene rings in the polymer chain. Furthermore, the signals at 753.4 and 695.5 were attributed to the out‐of‐plane bending vibration of the C−H group.^[81]^ Therefore, the IR spectra confirmed the chemical nature of PS microbeads. Moreover, the sediment showed similar peaks, proving the precipitation of the PS microbeads. On the other hand, the broad peak at 3351 cm^−1^ can be attributed to the stretching absorption of hydroxyl groups due to water bonded via hydrogen bonding, or to the hydrolysis product of FeCl_3_. Similar conclusion can be generalized for Figure 7b, where PET characteristic bands were detected, such as the symmetrical C−H stretching at 2966.6 cm^−1^, the C=O stretching of the carboxyl groups at 1717.3 cm^−1^, the aromatic C=C vibrations (1576.6 and 1502.2 cm^−1^), and the terephthalate group vibrations (1237.4 and 1092.6 cm^−1^).[^82^, ^83^] As in the case of PS, the same bands can be observed in the spectrum of the precipitated sample with a wider peak at about 3310.0 cm^−1^, while no similar peaks are detected in the supernatant. However, since there was no particular indication for the formation of new bonds, chemisorption processes probably did not occur in the CFS tests, and the obtained removal should be mostly charged on sweep flocculation. Indeed, this latter mechanism basically consists of the enmeshment of MPs operated by the formed flocs consisting of the FeCl_3_ hydrolysates, having the ability of capturing the suspended particles during the slow mixing phase drawing them to the bottom while settling.[^84^, ^85^] The formation and properties of the hydrolysates of Fe‐based coagulants are mostly dependent on pH.^[66]^ Nonetheless, the influence of this parameter over CFS performance was not the object of the present investigation. However, observing the formation of flocs during pH adjustment phases, it was noted that no visible flocs appeared at pH lower than 6.5, whereas for pH above 8.0 their growth required longer time. Therefore, keeping pH around 7 ensure the formation of flocs capable of capturing MPs of the considered sizes, as reported from Ma et al. for the removal of PE fragments.[^51^, ^52^]

### FeCl_3_‐SA and FeCl_3_‐CT Coagulation Performance

**Figure 8:**
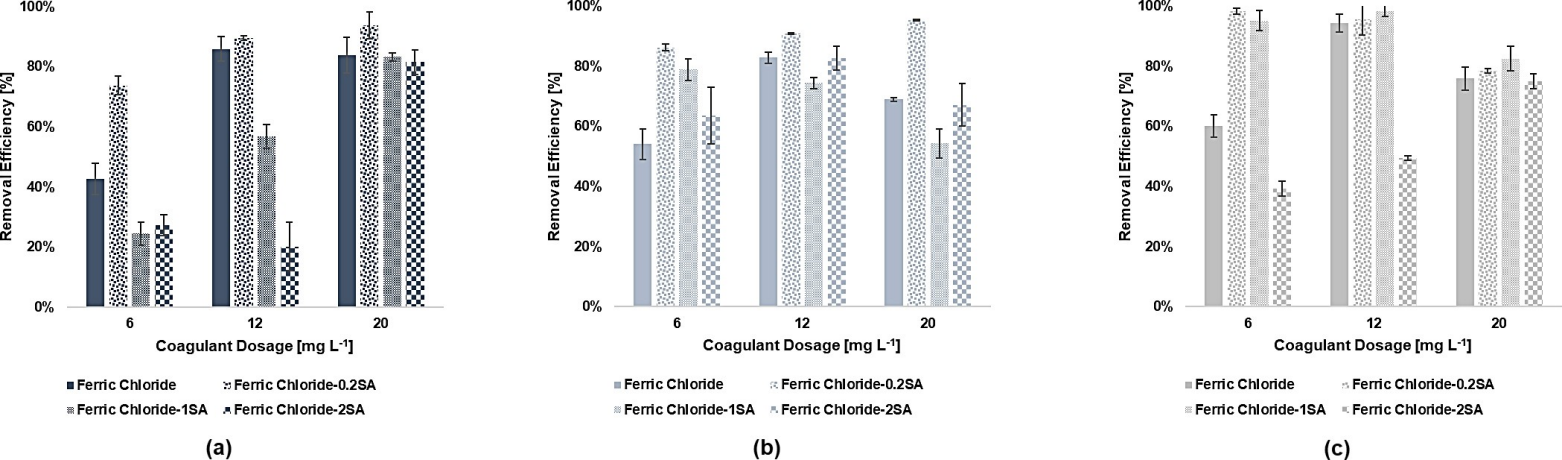
Removal efficiency of a) PS, b) PE, and c) PET microplastics under different FeCl_3_‐SA dosages. Initial pH 7, initial concentration of 300 mg L^−1^. Error bars represent SD from three independent replicates.

**Figure 9:**
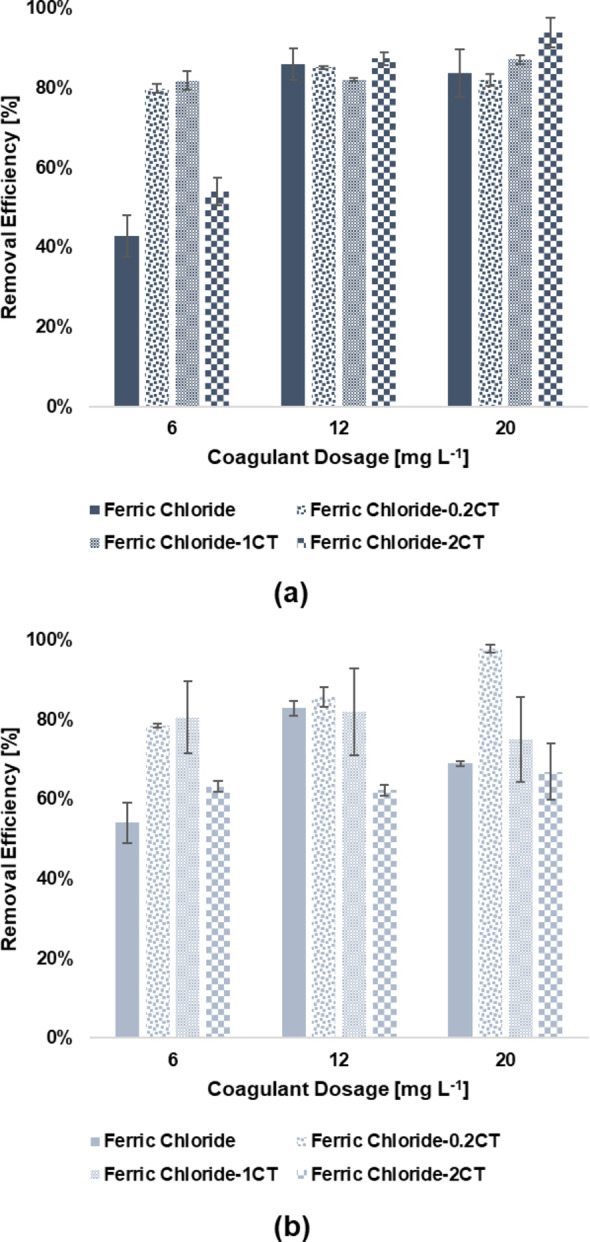
Removal efficiency of a) PS, and b) PE under different FeCl_3_‐CT dosages. Initial pH 7, initial concentration of 300 mg L^−1^. Error bars represent SD from three independent replicates.

**Figure 10:**
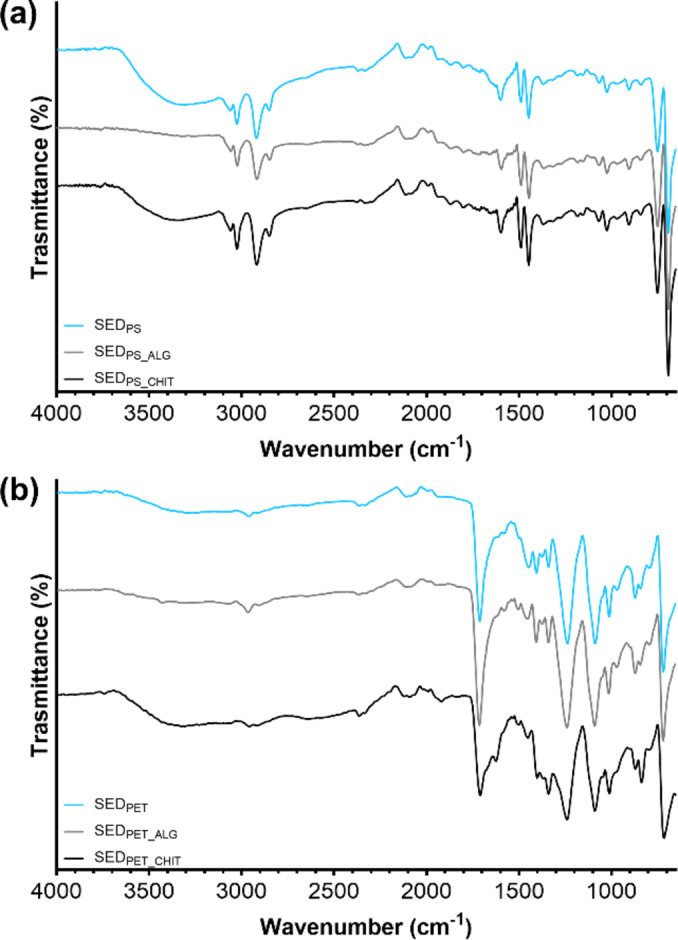
FTIR spectra of a) PS microbeads after CFS treatment with FeCl_3_, FeCl_3_‐SA, and FeCl_3_‐CT systems; b) PET fragments after CFS treatment with FeCl_3_, FeCl_3_‐SA, and FeCl_3_‐CT systems.

The experiments foreseeing the use of coagulant aids were performed in a narrower range of primary coagulant dosage (i. e., 6 to 20 mg L^−1^). The dosage of both SA and CT ranged from 0.2 mg L^−1^ to 2 mg L^−1^ and the resulting removal rates are shown in Figure 8 and Figure 9, respectively. With reference to Figure 8a, 8b, and 8c, the major effectiveness for the removal of PS, PE, and PET particles was linked to the use of the lowest amount of SA. Indeed, injecting 0.2 mg L^−1^ of SA with 12 mg L^−1^ of FeCl_3_ led to removal efficiencies of 89.53 %±0.69 %, 90.77 %±0.28 %, and 95.53 %±5.31 % for PS, PE, and PET, respectively. The statistical results made possible to identify a better option for PS, PE, and PET in decreasing the dosage of FeCl_3_ while injecting the minimal amount of SA. Indeed, comparing the combination of 6 mg L^−1^ of FeCl_3_ with 0.2 mg L^−1^of SA and the sole use of 12 mg L^−1^ of FeCl_3_ resulted to be not significant for all the tested MPs (PS, p=0.2211; PE, p=0.9983; PET, p=0.9543). In most cases, the increase in SA dosage led to a decrease in the efficiencies with respect to the FeCl_3_ system, though operating at high dosages of primary coagulant with high dosages of SA resulted beneficial in some cases. For instance, the removal rates of PS at the highest dosage of FeCl_3_ was roughly unaffected by the addition of 2 mg L^−1^ of SA (81.48 %±4.00 % versus 83.73 %±5.94 % of the base case), and similar evidence might be retrieved for PE and PET removal (PS, PE, and PET, p >0.9999). The measurement of the ζ potential partially confirmed the reason behind the loss of effectiveness at high dosages of SA, with values consistently lower than the ones obtained for the base case. Similar results were also obtained from Zhang et al., who noticed a reduction in ζ potential for the PAC‐SA system with respect to the PAC system, assuming that it could be prompt by the negative charges of the hydrolysates of SA. However, the authors reported that 5 mg L^−1^ of SA led to a 10 % increase in removal efficiency at conventional dosage of PAC, reporting a maximum efficiency of 73.35 % at PAC and SA dosages of 200 mg L^−1^ and 100 mg L^−1^, respectively.^[62]^ This suggests that SA primarily stabilizes flocs at low dosages but may counteract FeCl₃ hydrolysis products at higher concentrations, leading to suboptimal aggregation. Moreover, as can be seen from the IR spectra in Figure 10, there were no significant changes in the spectrum of the sediment for both PS and PET with respect to the FeCl_3_ system at SA dosage of 0.2 mg L^−1^. Nonetheless, literature findings highlight the chance of an enlargement of flocs and an enhancement of robustness driven by the interactions between SA and iron ions and hydrolysates,[^86^, ^87^] and this might indicate and confirm that sweep flocculation could be the most relevant mechanism underlying the removal of the selected MPs. As for the FeCl_3_‐CT, as reported in Figure 9a and 9b the use of 0.2 mg L^−1^ did not have significant effects on the removal of PS and PE at 12 mg L^−1^ of FeCl_3_, with the rates being stable (85.08 %±0.32 % versus 85.93 %±3.98 % of the base case, and 85.65 %±2.44 % versus 82.86 %±1.88 % of the base case for PS and PE, respectively). The scarce significance was also confirmed by the statistical analysis (PS and PE, p >0.9999) However, the addition of this low dosage of CT led to the improvement in performance for 6 mg L^−1^ of FeCl_3_, with efficiencies up to 79.79 %±1.12 % and 78.41 %±0.47 % for PS and PE versus rates of 42.74 %±5.21 % and 54.03 %±5.07 % in the base case, respectively. The obtained differences were highly significant for PS (p <0.0001) and significant for PE (p=0.0064) This indicates the chance of further reducing the amount of primary coagulant dosage. Despite the cationic nature of CT and its recognised ability to aggregate anionic soluble compounds through electrostatic affinities,^[88]^ ζ potential values poorly correlated with the enhancement in removal performance obtained. Investigating the effectiveness of FeCl_3_‐CT system to remove PS microsphere, Raji et al. note also reported a beneficial effect of CT with minimal dosages of primary coagulant, suggesting that the main driving force of the removal process might be the cross‐linking effect exerted by the polymer, capable of also overcoming sharp changes in ζ potential and potential charge reversal related issues.^[89]^ By contrast, the residual concentration of PET after FeCl_3_‐CT treatment increased just by adding 0.2 mg L^−1^ of CT at all the investigated dosages of primary coagulants. The worsening in removal performance has been partially related to the ζ potential turning out to be positive at all the investigated dosages potentially indicating an increased stabilization of the suspension. A further confirm came from the IR spectrum of the PET sediment (Figure 10b), where new peaks can be recognized at 1622.2 cm^−1^ and 1038.9 cm^−1^, attributable to the stretching of the C=O of the secondary amide and to the C−O bending vibration of chitosan.^[90]^ Furthermore, a broad signal between 1400–1300 cm^−1^ can be observed. This contribution was assigned to the C−N stretching of amide III, and to the CH_2_ bending and CH_3_ symmetrical deformations of the chitosan chain.^[91]^

These results are in line with the findings on ζ potential, revealing the great affinity of chitosan with PET fragments even at low concentration level, therefore confirming the involvement of the positively charged polymer in the reduced sedimentation of the PET. In practical terms, these findings emphasize the importance of precise coagulant aid dosing to balance removal efficiency with economic and environmental considerations. SA proves advantageous in stabilizing floc structures at low dosages, yet its negative charge effects at higher concentrations require careful control to avoid performance loss. Conversely, CT enhances bridging and aggregation at reduced FeCl₃ dosages, but its strong affinity for PET may lead to over‐stabilization, hindering sedimentation. From an operational perspective, SA stands out for its accessibility and cost‐effectiveness, whereas CT, despite its high removal efficiency, faces sourcing constraints and cost challenges due to its widespread use in biomedical and food industries.^[63]^

## Conclusions

MPs pollution remains a persistent threat to ecosystem health, while the existing impact mitigation strategies will require decades to yield tangible results. As MPs continue to accumulate, immediate action is essential to intercept them at their source. WWTPs serve as critical interfaces between human activities and the environment, requiring targeted advancements in sustainable technologies and renovation of existing facilities to enhance removal performance while ensuring strict compliance with evolving environmental and technical standards. Our research investigates the potential of introducing natural coagulant aids, such as CT and SA, to maintain or enhance the performance of conventional treatment systems while minimizing unintended ecological impacts downstream. By integrating eco‐design principles in both material selection and treatment infrastructure, we can establish a comprehensive, long‐term approach to safeguarding environmental and public health. The outcomes of the experiments on PS, PE, and PET particles proved that CT and SA led to an improvement in MPs removal with respect to the sole use of conventional iron‐based salts at low dosages. The highest removal rates were obtained in the case of 20 mg L^−1^ and 0.2 mg L^−1^ of FeCl_3_‐SA for PS (93.77 %), 20 mg L^−1^ and 0.2 mg L^−1^ of FeCl_3_‐CT for PE (97.81 %) mg L^−1^, and of 12 mg L^−1^ and 0.2 mg L^−1^ of FeCl_3_‐SA for PET (98.39 % removal). Nonetheless, the performed analyses demonstrated the chance of obtaining nearly identical performances by operating at a drastically lower dosage of primary coagulant (6 mg L^−1^) and by injecting 0.2 mg L^−1^ of coagulant aid. These results support the need to further investigate the cost reduction potential of moving to more sustainable coagulation systems. The investigation of some of the main coagulation mechanisms allowed us to conclude that, in the absence of coagulant aids, charge neutralization might be one driving force of the process alongside sweep flocculation. On the other hand, for FeCl_3_‐CT and FeCl_3_‐SA there was no evidence to support the prevalence of one mechanism to the others, though it is possible to assume that sweep flocculation and bridging played a major role. Future studies will be devoted to a more holistic understanding of the occurrence and significance of the mechanisms underlying the CFS process in the presence of natural coagulants and in more complex matrices. Notably, the application of natural coagulants must consider potential sludge management challenges, as previous studies suggest that excessive polymer dosage can lead to increased organic load in sludge, affecting disposal and treatment strategies.

## Conflict of Interests

The authors declare no conflict of interest.

1

## Data Availability

The data that support the findings of this study are available from the corresponding author upon reasonable request.
